# Enterovirus B types cause severe infection in infants aged 0–3 months

**DOI:** 10.1186/s12985-023-01965-9

**Published:** 2023-01-09

**Authors:** Xiaohan Yang, Lei Duan, Wenli Zhan, Yuan Tang, Lihua Liang, Jia Xie, Mingyong Luo

**Affiliations:** 1 Medical Genetic Center, Guangdong Women and Children Hospital, Guangzhou, 511400 China; 2 Department of Clinical Laboratory, Taian City Central Hospital, Shandong, 271000 China; 3 Department of Clinical Laboratory, Guangdong Women and Children Hospital, Guangzhou, 511400 China; 4 Guangzhou Medical University, Guangzhou, 511436 China

**Keywords:** Enterovirus B, Infant, Severe infection, Recombination

## Abstract

**Background:**

Enterovirus (EV) infections are being increasingly seen in younger infants, often being more severe than in older children. The risk factors of EV infection in infants have been inadequately investigated till date.

**Methods:**

We conducted a retrospective study on hospitalized children with laboratory-confirmed EV infection (50 infants aged 0–3 months and 65 older than 3 months) at a tertiary care center in China. Prevalence, clinical characteristics, and genetic features of the virus were analyzed, and independent predictors for severe infection were assessed.

**Results:**

Clinical findings showed that severe infection was more common in infants aged 0–3 months than in older children (78.0% vs. 35.4%, *p* < 0.001), with higher morbidity of pneumonia, meningitis, and sepsis (*p* < 0.01). EV-B types were detected more frequently in infants aged 0–3 months than in older children (88.0% vs. 7.7%, *p* < 0.001). Echovirus 11 was the most identified EV-B, and it recombined with E6 in P2 and P3 regions. Risk factors for severe EV infection included EV-B types infection, age less than 3 months, elevated alanine aminotransferase level, abnormal platelet count, and abnormal cerebrospinal fluid characteristics.

**Conclusions:**

Our data indicated that EV-B types mainly cause severe infection in infants aged 0–3 months. Therefore, knowledge about EV-B types could have implications in designing effective intervention and prevention strategies for young infants with severe EV infection.

**Supplementary Information:**

The online version contains supplementary material available at 10.1186/s12985-023-01965-9.

## Background

Enterovirus (EV) are small non-enveloped RNA viruses belonging to the family *Picornaviridae*, with genomes approximately 7500 nucleotides in length. Currently, more than 100 EV types, assigned to four species, have been found to infect humans, namely enterovirus A (EV-A), EV-B, EV-C, and EV-D, based on genetic divergence [1, 2]. EV are distributed worldwide and have a seasonal incidence patterns in temperate regions during summer and fall and occur year-round in the tropics [3]. EV infection in children manifest as a spectrum of clinical disorders, including non-specific febrile illness, hand-foot-mouth disease (HFMD), meningitis, viral encephalitis, myocarditis, sepsis, and pulmonary edema. The severity and mortality of EV infection are generally inversely related to age, especially in newborns within the first few days of life [4, 5].

In recent decades, several EV-A-associated HFMD outbreaks, particularly those caused by EV-A71, coxsackievirus (CV) A6, CVA10, and CVA16 types, involving millions of children under 5 years of age, have been described in detail [6–9]. Unlike EV-A infections, EV-B infections (echovirus (E) 1–7, E9, E11–21, E24–27, E29–33, CVB1–6, and CVA9) are commonly identified in children less than 1 year old and are characterized by serious disease, with high mortality rate [10, 11]. The circulation of EV-B types has been associated with several EV outbreaks in Asia, Europe, and the USA in recent years [12–14]. However, the incidence of infant infections and circulating types varies markedly across countries and regions.

There is growing interest in the understanding of epidemiology of EV in infants, particularly due to their association with severe disorders. Awareness of the clinical features associated with severe conditions in infants, recognition of the risk factors, and monitoring of the infection types might help pediatricians diagnose severe cases promptly and treat them appropriately to reduce mortality. In this study, we described the epidemiological, clinical, and genetic characteristics of EV infections in a cohort of children admitted to a tertiary care center in Guangzhou, China. Furthermore, with the clinical and molecular epidemiological patterns of EV infection, we aimed to explore the clinical spectrum and relationship of individual EV types with the risk factors of severe infection in infants.

## Methods

### Study design and patients

Children hospitalized with laboratory-confirmed EV infection, between January and December 2019, at the Guangdong Women and Children Hospital, Guangzhou, China were included in this study. A confirmed case of EV infection was defined by positive EV-RNA findings in stool, plasma, and/or cerebrospinal fluid (CSF) specimens. Moreover, we extracted demographic data, clinical symptoms or signs, laboratory data, CSF parameters, and outcomes upon admission from the patients’ electronic medical records, and all laboratory tests were performed according to the clinical care needs of the children.

### EV detection and type determination

Nucleic acids were extracted from stool, plasma, and/or CSF samples of suspected children using the Ex-DNA/RNA Virus Kits (Tianlong Bio-technology, Suzhou, China), and amplified using the commercial pan-enterovirus q-PCR kit (DAAN Gene, Guangzhou, China) for EV in the 7500 fast real-time PCR system (Applied Biosystems, Foster City, USA), according to the manufacturer’s instructions [15]. In addition, all EV-positive samples were amplified on the basis of partial VP1 sequences for further type identification, as described previously [15].

### Full-length genome amplification

To understand the molecular epidemiology of EV in infants better, three long-distance PCR amplifications of the most common EV type in infants were obtained using the TaKaRa LA Taq^®^ Kit (Dalian, China). The primers used for RT-PCR are listed in Additional file 1: Table S1, and the full-length genome was sequenced using the “primer-walking” strategy. Finally, complete nucleotide sequences of selected strains were obtained by assembling the sequenced fragments, which were eventually submitted to GenBank (accession numbers: MW883610 to MW883614).

### Phylogenetic and recombination analysis

Multiple sequence alignments were performed using the ClustalX, and phylogenetic trees were drawn using the neighbor-joining method via MEGA X [16]. Bootstrap analyses with 1000 re-samplings were performed to determine the confidence values for groupings within the phylogenetic trees. The SimPlot and Bootscan analyses were performed using SimPlot 3.5.1 with a 500-nucleotide window moving in steps of 20 nucleotides [17]. Evolutionary divergence of the 5ʹ UTR, four viral capsid proteins (VP1–VP4), and seven non-structural proteins (2A–2C and 3A–3D) between the selected strains and 64 reference strains from the GenBank was estimated via MEGA X using the Kimura 2-parameter model [16]. Heatmap of the 5ʹ UTR, VP1–VP4, 2A–2C, and 3A–3D genes was created based on evolutionary divergences.

### Statistical analysis

Descriptive statistical analysis for population characteristics in the study and clinical findings was performed using SPSS 22.0. Categorical variables are reported as frequencies and percentages after comparison using the chi-square test or Fisher’s exact test. The quantitative data are presented as the median (interquartile range, IQR) and were compared using the nonparametric rank-sum test (Kruskal–Wallis test). Odds ratio (OR) and 95% confidence intervals (95% CIs) were used to estimate the risk factors of severe infection using SPSS 22.0, a multiple logistic regression analysis.

## Results

### Demographic characteristics and seasonal distribution

**Fig. 1:**
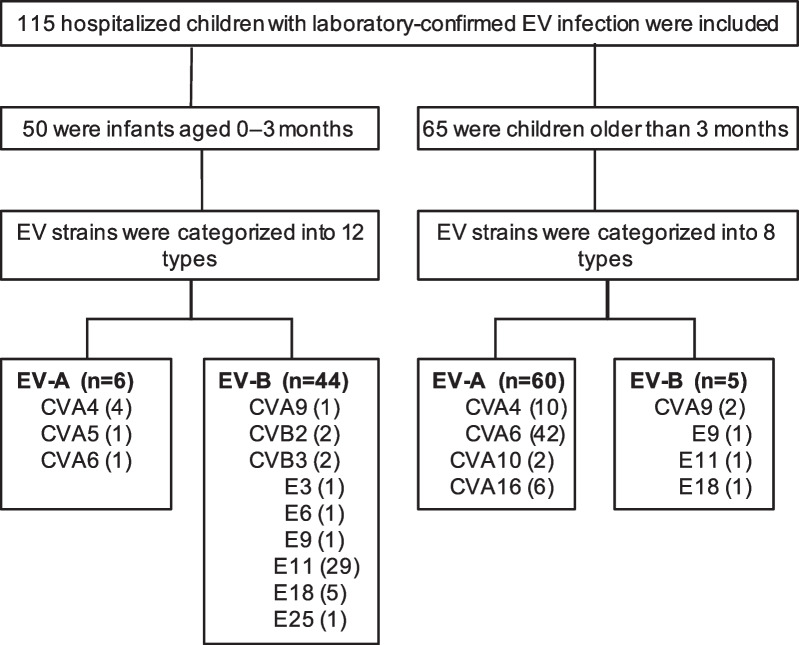
Flow diagram of children hospitalized with EV infections in Guangzhou, China during January–December 2019

A total of 115 children, hospitalized with laboratory-confirmed EV infections between January and December 2019, were included in the study (Fig. 1). The median age was 10.0 months (IQR 0.7–19.0), and 75 (65.2%) participants were males. Fifty (43.5%) participants were infants aged 0–3 months [median age 21 days (IQR 12–27)], and 65 (56.5%) were children older than 3 months [median age 17.0 months (13.0–26.5)].

**Fig. 2:**
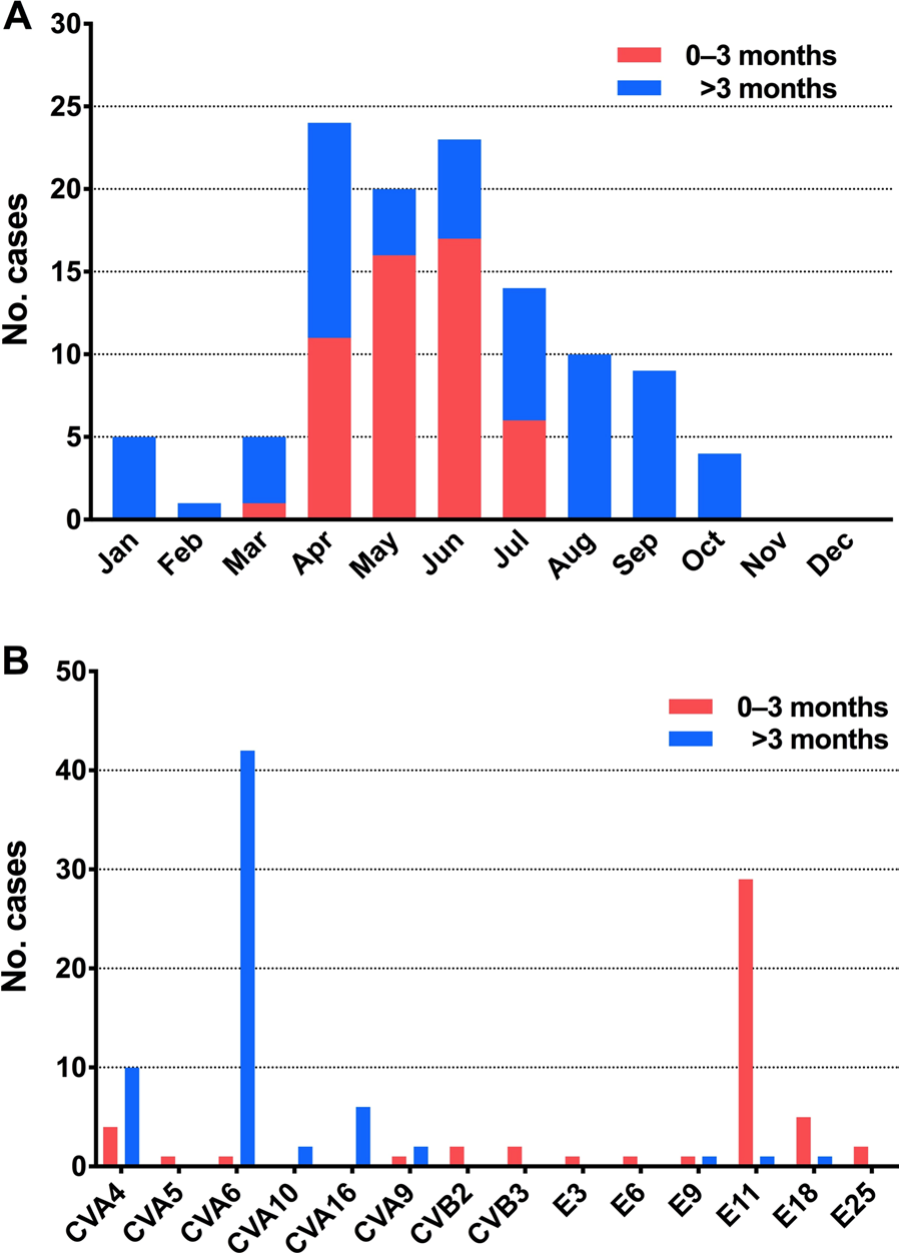
Children hospitalized with EV infections, as per age group, month, and EV types.** a** Number of children hospitalized with EV infections, by age group and month.** b** Number of children hospitalized with EV infections by age group and EV types

Since the incidence and types of EV infection are influenced by seasonal cycles, we checked the monthly variation (Fig. 2a). A total of 109 (94.8%) hospitalizations occurred from March to October, and the number of cases peaked from April to June. However, there was a significant difference in seasonal changes in the occurrence of EV infection between infants aged 0–3 months and children older than 3 months. A peak (mainly caused by E11) was observed in the infant group from April to July, with no case thereafter. On the contrary, two peaks were observed in the number of cases in the children group. The first peak was unexpected, appearing in April; the second peak appeared in August.

### Type distribution

Two EV species, comprising of 14 different types, accounted for all the 115 reports over this period. EV-A types, including CVA6 [43 (37.4%) of 115], CVA4 [14 (12.2%)], CVA16 [6 (5.2%)], CVA10 [2 (1.7%)], and CVA5 [1 (0.9%)], were identified in 66 (57.4%) cases. EV-B, accounting for the remaining 49 (43.6%) cases, included E11 [30 (26.1%)], E18 [6 (5.2%)], CVA9 [3 (2.6%)], E25 [2 (1.7%)], E9 [2 (1.7%)], CVB3 [2 (1.7%)], CVB2 [2 (1.7%)], E3 [1 (0.9%)], and E6 [1 (0.9%)]. The median age of EV-B infections was 21 days (IQR 12–27), which was significantly less than the 16.5 months (IQR 13.0–26.0) observed for EV-A infections (*p* < 0.001).

The species of EV infecting at different ages were significantly different (Fig. 2b). EV-B was detected more frequently in infants aged 0–3 months than in older children [44 (88.0%) of 50 vs. 5 (7.7%) of 65, *p* < 0.001]. However, EV-A was more common in children older than 3 months [60 (92.3%) of 65 vs. 6 (12.0%) of 50, *p* < 0.001], and only 5 (7.7%) were infected with EV-B. E11 was the predominant serotype identified in 29 (58.0%) of the 50 infants, with a noticeably higher proportion than that in older children [1 (1.5%) of 65] (*p* < 0.001). Additionally, E18 was detected in five (10.00%) infants, whereas only one such case (1.5%) was found in older children. However, CVA6 was the most commonly observed serotype in older children [42 (64.6%) of 65], followed by CVA4 [10 (15.4%)] and CVA16 [6 (9.2%)].

### Clinical analysis

Clinical case files of the 115 patients, with symptoms registered by the pediatrician upon hospital admission, were carefully analyzed, according to age and EV species (Table 1 and Additional file 2: Table S2). The patients showed typical signs and symptoms, including fever [103 (89.6%) of 115], rash [60 (52.2%)], tachycardia [40 (34.8%)], coughing [37 (32.2%)], convulsions [28 (24.3%)], and diarrhea [23 (20.0%)]. However, presentation with fever, rash, tachycardia, coughing, vomiting, startle, and convulsions was significantly less common among infants aged 0–3 months than in children older than 3 months (all *p* < 0.01). Although the clinical features of infants aged 0–3 months were not typical, the median days of hospital stay were significantly longer than those of older children [13 (IQR7–17) vs. 5 (IQR 4–7), *p* < 0.001].

**Table 1:** Clinical characteristics of children with EV infection according to age, Guangzhou, China, 2019

	All cases, n = 115	0–3 months, n = 50	> 3 months, n = 65	*p*
Age (months)	10.0 (0.7–19.0)	0.7 (0.4–0.9)	17.0 (13.0–26.5)	N.D
Male sex	75 (65.2%)	29 (58.0%)	46 (70.8%)	N.S
Days of hospitalization	6 (4–13)	13 (7–17)	5 (4–7)	< 0.001
*Clinical symptoms*				
Fever (≥ 38℃)	103 (89.6%)	40 (80.0%)	63 (96.9%)	0.003
Rash	60 (52.2%)	4 (8.0%)	56 (86.2%)	N.S
Tachycardia^a^	40 (34.8%)	10 (20.0%)	30 (46.2%)	0.004
Lethargy	2 (1.7%)	1 (2.0%)	1 (1.5%)	N.S
Coughing	37 (32.2%)	8 (16.0%)	29 (44.6%)	0.001
Diarrhea	23 (20.0%)	7 (14.0%)	16 (24.6%)	N.S
Vomiting	15 (13%)	1 (2.0%)	14 (21.5%)	0.002
Pruritus	6 (5.2%)	0 (0.0%)	6 (9.2%)	N.S
Startle	12 (10.4%)	0 (0.0%)	12 (18.5%)	0.001
Convulsions	28 (24.3%)	2 (4.0%)	26 (40%)	< 0.001
Impaired consciousness	7 (6.1%)	5 (10.0%)	2 (3.1%)	N.S
Hand-foot-mouth disease	59 (51.3%)	1 (2.0%)	58 (89.2%)	< 0.001
Pneumonia	44 (38.3%)	34 (68.0%)	10 (15.4%)	< 0.001
Aseptic meningitis	36 (31.3%)	28 (56.0%)	8 (12.3%)	< 0.001
Encephalitis	6 (5.2%)	3 (6.0%)	3 (4.6%)	N.S
Sepsis	19 (16.5%)	14 (28.0%)	5 (7.7%)	0.004
Myocarditis	11 (9.6%)	8 (16.0%)	3 (4.6%)	N.S
Gastrointestinal dysfunction	23 (20%)	11 (22.0%)	12 (18.5%)	N.S
Hepatitis	8 (7.0%)	6 (12.0%)	2 (3.1%)	N.S
Pulmonary edema	3 (2.6%)	1 (2.0%)	2 (3.1%)	N.S
Severe infection	62 (53.9%)	39 (78.0%)	23 (35.4%)	< 0.001
Number of deaths	2 (1.7%)	1 (2.0%)	1 (1.5%)	N.S

^a^Tachycardia: age 0–3 months old, heart rate ≥ 140 times per minute, 4–12 months old, heart rate ≥ 130 times per minute, 1–3 years old, heart rate ≥ 120 times per minute, 4–7 years old, heart rate ≥ 100 times per minute, 8–14 years old, heart rate ≥ 90 times per minute

*N.S.* No significant; *N.D.* Not detected

The disease spectrum of patients with EV infection varied in this study. HFMD [59 (51.3%) of 115], pneumonia [44 (38.3%)], meningitis [36 (31.3%)], sepsis [19 (16.5%)], and gastrointestinal dysfunction [23 (20.0%)] were the main disorders frequently observed in 115 patients. Of the 59 patients who fulfilled the definition of HFMD, 55 (93.2%) cases were caused by EV-A, and four (6.8%) were caused by EV-B. Majority [58 (98.3%) of 59] of HFMD cases were children older than 3 months, and only one was an infant. However, the infants aged 0–3 months had significantly higher prevalence rates of pneumonia [34 (68.0%), 50 vs. 10 (15.4%) of 65, *p* < 0.01], meningitis [28 (56.0%) vs. 8 (12.3%), *p* < 0.001], and sepsis [14 (28.0%) vs. 5 (7.7%), *p* < 0.001] than older children. Hepatitis occurred in six (5.2%) infants and two (1.7%) children, and all the cases were associated with EV-B infections.

A total of 62 (53.9%) cases were classified as having severe disease, of whom 39 (62.9%) were infants. Thus, the incidence of severe EV-B-associated infection was 73.5% (36 of 49), which was significantly higher than the 39.4% (26 of 66) of EV-A infections (*p* < 0.001). Notably, two EV-B-associated individuals died after the onset of the disease; one 5-day-old infant was infected with CVB3, and died of pulmonary edema and subventricular hemorrhage after 28 days of ventilator support, and another (7-month-old) was infected by E18, and died of brain edema.

### Laboratory parameters of blood and CSF

Blood test results, according to age and EV serotypes, are presented in Table 2. Compared to older children, infants aged 0–3 months had higher neutrophil counts, lymphocyte counts, and lactate dehydrogenase (LDH) levels (all *p* < 0.05). Additionally, the levels of inflammatory markers like C-reaction protein (CRP) in older children were significantly higher than those in the infant group [15.3 mg/L (IQR 4.7–31.5) vs. 3.5 mg/L (1.0–18.5), *p* = 0.004].

**Table 2:** Laboratory tests from the patients with EV infection, Guangzhou, China, 2019

Blood characteristics	All cases	0–3 months	> 3 months	*p*
	n = 115	n = 50	n = 65	N.D
WBC (3.5–9.5 × 10^9^/L)	11.0 (7.4–13.8)	11.1 (8.6–13.9)	10.7 (7.2–13.3)	N.S
NEUT (1.8–6.3 × 10^9^/L)	5.4 (3.1–8.6)	4.3 (2.7–6.9)	6.2 (3.5–9.5)	0.048
LYMPH (1.1–3.2 × 10^9^/L)	3.3 (2–4.8)	4.2 (2.8–5.6)	2.6 (1.6–3.8)	0.006
PLT (125–350 × 10^9^/L)	315 (255–403)	333 (261–432)	308 (249–383)	N.S
PCT (0–0.1 ng/mL)	0.2 (0.1–0.6)	0.2 (0.1–0.8)	0.2 (0.1–0.5)	N.S
CRP (0–10 mg/L)	10.1 (2.5–23.9)	3.5 (1.0–18.5)	15.3 (4.7–31.5)	0.004
ALT (9–40 U/L)	25 (18–33)	28.5 (21–42)	24 (17–31)	N.S
AST (15–40 U/L)	37 (31–49)	38 (30–57)	37 (32–47)	N.S
UA (208–428 μmol/L)	202 (154–304)	170 (133–212)	250 (190–337)	0.007
LDH (313–618 U/L)	629 (326–774)	716 (375–1075)	587 (299–695)	0.002
CK (40–200 U/L)	86 (63–141)	84 (60–115)	97 (64–159)	N.S
GLU (3.3–5.6 mmol/L)	5.8 (5.0–6.9)	5.8 (4.9–7.8)	5.9 (5.1–6.5)	N.S

CSF characteristics	All cases	0–3 months	> 3 months	*p*
	n = 60	n = 33	n = 27	N.D
WBC	3.0 (1.0–16.0)	8.0(3.0–48.0)	1.0 (0–2.5)	< 0.001
Pleocytosis (%)	30.0 (18/60)	42.4 (14/33)	14.8 (4/27)	0.020
Protein (mg/dL)^a^	494 (167–935)	921 (629–1084)	151 (100–212)	< 0.001
Elevated protein (%)	35.0 (21/60)	63.6 (21/33)	0	< 0.001
LDH (U/L)	100 (60–124)	104 (50–149)	100 (100–104)	N.S
Cl^−^ (mmol/L)	121 (119–124)	122 (120–123)	121 (118–124)	N.S
Glucose (mg/dL)	2.9 (2.3–3.4)	2.4 (1.9–2.9)	3.3 (3.1–3.8)	< 0.001
Low CSF glucose (%)	41.7 (25/60)	69.7 (23/33)	7.4 (2/27)	< 0.001
Positive rate of EV (%)^b^	84.5 (153/181)	91.2 (83/91)	77.8 (70/90)	0.012
Stool (%)	100 (115/115)	100 (50/50)	100 (65/65)	N.S
Plasma (%)	90.9 (10/11)	90.9 (10/11)	0	N.D
CSF (%)	50.91 (28/55)	76.7 (23/30)	20.0 (5/25)	< 0.001

^a^CSF protein concentration was classified as normal if it was ≤ 900 mg/dL for newborn babies (aged ≤ 28 days) and ≤ 450 mg/dL for older children (aged > 28 days); ^b^EV-RNA detected in stool, plasma, and CSF samples by fluorescence PCR

*EV* Enterovirus; *WBC* White blood cell; *NEUT* Neutrophils; *LYMPH* Lymphocytes; *PLT* Platelets; *PCT* Procalcitonin; *CRP* C-reactive protein; *ALT* Alanine aminotransferase; *AST* Aspartate aminotransferase; *UA* Uric acid; *LDH* Lactate dehydrogenase; *CK* Creatine kinase; *GLU* Glucose; *CSF* Cerebrospinal fluid; *Cl* Chloride; *N.S.* No significant; *N.D.* Not detected

CSF parameters according to EV type and age are shown in Table 2. In this study, a total of 60 CSF samples from 33 infants (aged 0–3 months) and 27 older children were included. CSF pleocytosis, elevated protein levels, and low CSF glucose were significantly more common in infants than in children (all *p* < 0.001). In addition, the positive rate of EV in CSF samples from young infants was 76.7% (23 of 30), which was significantly higher than the 20.0% (5 of 25) in the children group (*p* < 0.001).

### Risk factors for severe infection

In this study, among the 115 patients with EV infections, 62 (53.9%) developed severe disease. Risk factors were analyzed between severe and non-severe EV infections by multiple logistic regression analysis using the forward stepwise logistic regression model. As shown in Table 3, severe EV infection was associated with the following factors: infection by EV-B (OR 4.260, 95% CI 1.907–9.517), young age, less than 3 months (OR 6.474, 95% CI 2.794–15.002), abnormal platelet count (OR 2.745, 95% CI 1.278–5.897), and ALT level > 40 U/L (OR 3.064, 95% CI 1.031–9.105). Additionally, abnormal CSF characteristics, including EV positivity (OR 12.071, 95% CI 2.379–61.261), elevated protein (OR 13.913, 95% CI 1.691–114.447), and pleocytosis (OR 9.481, 95% CI 5.633–27.413) were also independent predictors of severe infection.

**Table 3:** Multinomial logistic regression analysis of risk factors for severe EV infection

Factors	*β*-coefficient	OR	95%CI	*p*
EV-B infection	1.449	4.26	1.907–9.517	< 0.001
Age less than 3 months	1.868	6.474	2.794–15.002	< 0.001
Abnormal platelets count	1.01	2.745	1.278–5.897	0.010
Elevated ALT	1.12	3.064	1.031–9.105	0.044
*CSF characteristics*				
Positive of EV^a^	2.491	12.071	2.379–61.261	0.003
Elevated protein	2.633	13.913	1.691–114.447	0.014
Pleocytosis	2.249	9.481	5.633–27.413	0.037

^a^EV-RNA detected by fluorescence PCR

*OR* Odds ratio; *CI* Confidence interval; *EV* Enterovirus; *ALT* Alanine aminotransferase; *CSF* Cerebrospinal fluid

### Phylogenetic analysis of EV-B types

**Fig. 3:**
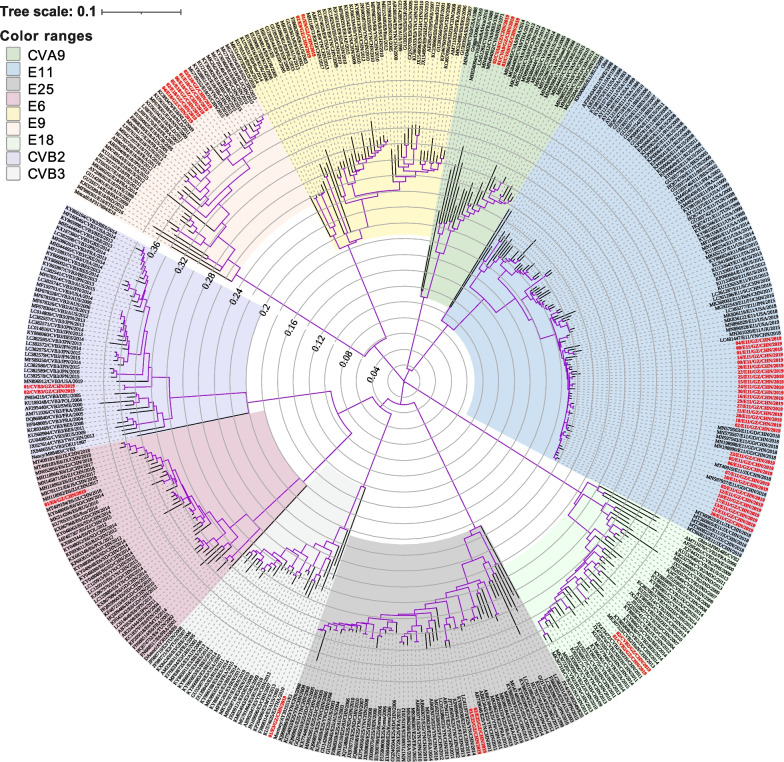
Neighbor-joining tree of the partial VP1 gene of the EV-B strains in Guangzhou, China. The 44 EV-B strains obtained from infants aged 0–3 months and 5 EV-B strains obtained from children older than 3 months, in this study, are shown in red. Scale bar indicates the number of nucleotide substitutions per site. Bootstrap values were calculated on 1000 replicates. Phylogenetic nodes with bootstrap values over 80 are marked as purple lines

To understand the molecular epidemiology of EV-B types better, a portion of the VP1 gene from all 49 viruses isolated from EV-B infections was amplified and selected for phylogenetic analysis (Fig. 3). The phylogenetic tree indicated that all 30 E11 strains were closely related to viruses detected in China and the USA in 2018. Additionally, all six E18 strains showed high homology with the strains isolated from the Yunnan, Jiangsu, Hebei, and Sichuan provinces of China in 2015 and 2016.

### Complete genome sequence analysis of E11

To help determine the evolution of predominant EV type in infants aged 0–3 months during the study year, we randomly performed full-length genome sequencing of E11 from five infants. Phylogenetic analysis revealed that all five E11 strains that circulated in Guangzhou city during 2019 clustered monophyletically with the E11 strains (MN597937 and MN597943) isolated from sewage samples in 2018 in Guangzhou city.

**Fig. 4:**
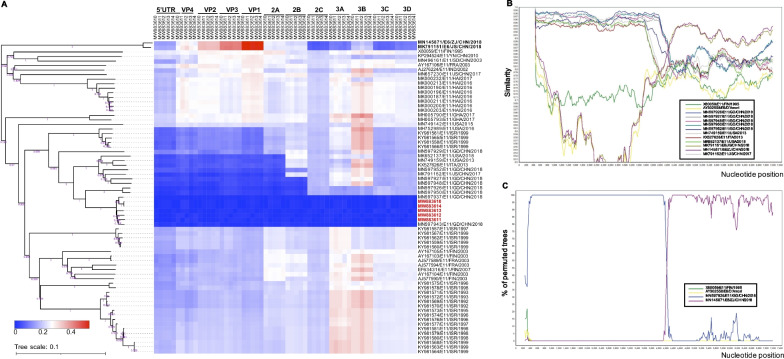
Recombination analysis of E11 genomes in Guangzhou, China during January–December 2019. **a** Heatmap representation of evolutionary divergences between 64 reference strains and 5 E11 strains (MW883610–MW883614) obtained. Scale bars indicate the number of nucleotide substitutions per site, and bootstrap values were calculated on 1000 replicates. Red squares show positive correlation, and blue squares show negative correlation across the different regions of E6 and E11. **b** Similarity plot analysis comparing the 5 E11 strains (MW883610–MW883614), obtained in this study, with other closely related EV-B reference strains using Simplot software version 3.5.1 with a window size of 500 nt and step size of 20 bp. **c** Bootscan analysis comparing 5 E11 strains (MW883610–MW883614) obtained in this study with other closely related EV-B reference strains, using a window size of 500 nt and step size of 20 bp, and genetic distance of the Kirmura 2-parameter model. The arbitrary recombinant threshold was 70%

Based on the heatmap results of evolutionary divergence between E11 sequence and other closely related EV sequences, we found that the nucleotide sequences of 5ʹ UTR, VP1–VP4, 2A, and 2B regions of E11 strains displayed higher sequence identity with the strains (MN597927, MN597948, MN597926, and MN597950) isolated in Guangzhou city in 2018, whereas 2C and 3A–3D genes showed minimum evolutionary divergence with E6 strains from Jiangsu province (MK791151) and Zhejiang province (MN145871) of China in 2018 (Fig. 4a). The highest similarity of 2C and 3A–3D regions with E6 suggested possible recombination.

Similarity plot and bootscanning were conducted between E11 and E6 strains to investigate the recombination phenomenon. Simplot analyses (Fig. 4b) showed that all five E11 strains in our study displayed the highest degree of identity with four E11 strains (MN597927, MN597948, MN597926, and MN597950) in the 5ʹ UTR and P1 (VP1–VP4) regions, and that similarity in the P2 (2B–2C) region decreased sharply. Thus, it was evident that recombination events with the E6 strain (MN597937 and MN597943) occurred partially in the P2 and P3 genomic regions. Furthermore, the Bootscan plot (Fig. 4c) indicated E11 strains in our study had a closer evolutionary relationship with E6 strains in the P2 and P3 genomic regions, with a sequence similarity of over 70.0%.

## Discussion

This study showed that seasonal variations in EV infections with respect to age were evident. Most infants aged 0–3 months had a seasonal pattern of infections. They presented during summer, with a peak from April to July, which was apparently different from the epidemic features in older children, and consistent with previous studies [4, 11, 18]. Our current study also showed that the circulating EV types, affecting each age range, differed substantially. The most common EV detected in children older than 3 months was EV-A, whereas EV-B, represented by the E11 type, was significantly more frequent in younger infants. In the USA and Europe, EV-B is the one most commonly reported in neonates [12, 13, 19, 20].

EV infection in neonates can present with clinical symptoms, ranging from asymptomatic, non-specific febrile illness to severe, life-threatening disease, and it is difficult to distinguish from non-EV infection based on the clinical signs only. In this study, fever was the most common symptom in both infants aged 0–3 months and older children. However, the other clinical symptoms, including rashes, tachycardia, coughing, vomiting, startle, and convulsions, frequently observed in children with HFMD, were not typical for infants, as suggested in earlier retrospective studies [13, 21]. Rashes are often suggested as a diagnostic basis for viral infection. However, only 6.0–27.3% of cases of infants with EV infection have been reported with rashes [4, 13, 22]. In this study, only 8% (4/50) of the infants developed cutaneous rashes. As an important indication of host inflammation in viral infection, less frequent rashes make the early clinical diagnosis of EV infection difficult for neonatologists.

Severe illnesses due to EV are commonly seen in neonates; mortality is exceptionally high when meningitis, myocarditis, and hepatitis occur [4, 13, 23]. More than three-quarters of neonates were diagnosed with severe infection in this study, particularly in the first two weeks of life. Meningitis, sepsis, pneumonia, myocarditis, and/or hepatitis are the most common clinical presentations. In contrast, only 35.4% of children older than 3 months showed severe infection. Meningitis is often associated with age-specific pleocytosis and/or neurological symptoms. Previous studies had shown that EVs can invade the central nervous system and disrupt the blood–brain barrier, resulting in more than 75% of meningitis cases, some of which are life-threatening [7, 13, 24]. In our study, out of 36 patients with meningitis, 28 (77.8%) were infants aged 0–3 months. It could be due to the immature brain of neonates and their imperfect blood–brain barrier. A higher detection rate of 76.7% (23 of 30) for EVs in CSF samples available from 30 neonates further substantiated this finding.

Pneumonia is the most common comorbidity in lower respiratory tract-EV-B infection, and can be rapidly progressive, leading to severe pneumonia [25]. In this study, more than one-third (44/115) cases were accompanied by pneumonia. EV-B types were the predominant pathogens in patients with pneumonia. Sepsis is a life-threatening organ dysfunction caused by a dysregulated host response to infection associated with a wide range of pathogens, and pediatricians frequently encounter it in young infants [13, 19, 26]. Hepatitis is often associated with EV-B infections, and mortality is especially high when it occurs concomitantly with myocarditis [27]. In this study, a total of 8 cases (6 infants and 2 children) developed symptoms of hepatitis during hospitalization, and all were associated with EV-B infection.

Understanding the risk factors and monitoring the parameters associated with severe infection may lead to effective prophylaxis and prompt aggressive treatment to reduce morbidity and mortality. Infection with EV-B and younger age are considered the major risk factors for developing severe infections [5]. A retrospective study on 2356 infants with known EV types during 1983–2003 reported that CVB1–5 and E11 have an increased risk of neonatal infection, with CVB4 being associated with the highest mortality rate of 40% [28]. In our study, we found that EV-B infection and age less than 3 months increased the risk of EV infection 4.260-fold and 6.474-fold, respectively. Moreover, of the two deceased children, one was positive for CVB3 and the other was for E18, which illustrated that EV-B infection is a major risk factor. Notably, the mother of the 7-month-old child was also positive for E18 as per stool sample test, with 100% similarity in VP1 gene compared to that of the children, but showed no obvious clinical symptom. Previous studies had shown that transmission of EVs from mother to infant is relatively common, occurring in 30–50% of cases [23, 29, 30].

Xiao-Qing Lv et al*.* had previously reported that an abnormal platelet count could be an independent predictor of severe EV infection [31]. Our results also showed that if the platelet count was abnormal, the risk of severe infection was increased 2.745-fold. Furthermore, abnormal CSF test result, such as pleocytosis, constituted a significant risk factor for developing severe EV infection, as observed by previous investigators [32], and elevated protein levels and pleocytosis in CSF increased the risk of severe infection 13.913-fold and 9.481-fold, respectively. Moreover, we elucidated that the positive result of EV in CSF was an independent predictor of severe infection, and the OR increased to 12.071.

Genomic recombination is a major driving force in the evolution, diversification, and shaping of genetic architecture of EVs [33], and time-correlated recombination events of EV-B are more frequent than those of other human EV species [34]. However, until recently, only limited complete genome sequences of E11 strains were available in the public database, with most of them coming from non-clinical isolates. In this study, we obtained 5 full-length E11 genomes, analyzed their phylogenetic characteristics, and found homologous recombination events to have occurred with E6 strains in China in 2018. Multiple phylogenetic studies presented previously provided evidence that RNA recombination of EVs only occurred throughout the entire non-structural region, and recombination sites were mainly located in region P2 [33–36]; notably, the recombination site was in the junction between 2B and 2C, as per our study. These observations suggested that non-structural proteins may be functionally interchangeable with other variants within EVs. Furthermore, in the infected host, effective recombination events are critical for RNA viruses to overcome tissue-type specific antiviral selection, establish robust infection and virulence, and adapt rapidly to dynamic selective environments [37, 38]. However, the changes in phenotypic characteristics of E11 recombination, including their fitness and pathogenicity, need to be investigated further.

## Conclusions

In summary, EV affects infants aged 0–3 months differently and more severely than in older children. Clinical manifestations in infants with EV mainly included meningitis, sepsis, pneumonia, and even death. EV-B types were the most common in neonatal EV infection, and recombination events were observed in the P2 and P3 regions of predominant type E11 with E6 from China. In addition, we identified independent predictors of severe EV infection, including EV-B infection, age less than 3 months, elevated ALT level, abnormal platelet count, and abnormal CSF characteristics. Taken together, EV-B infections should be routinely considered in neonates with meningitis, sepsis, and pneumonia, with or without a rash, particularly during EV season.

## Supplementary Information

**Additional file 1: Table S1.** Primers used for complete genome amplification of echovirus 11

**Additional file 2: Table S2.** Clinical characteristics of children with EV infection according to EV species, Guangzhou, China, 2019

## Data Availability

All data supporting the conclusions of this article are included herein.

## Abbreviations

EV: Enterovirus
CV: Coxsackievirus
E: Echovirus
HFMD: Hand-foot-mouth disease
CSF: Cerebrospinal fluid
OR: Odds ratio
CI: Confidence interval
GLU: Glucose
PCT: Procalcitonin
WBC: White blood cell
NEUT: Neutrophils
LYMPH: Lymphocytes
PLT: Platelets
CRP: C-reactive protein
ALT: Alanine aminotransferase
AST: Aspartate aminotransferase
UA: Uric acid
LDH: Lactate dehydrogenase
CK: Creatine kinase
Cl: Chloride
VP: Viral capsid protein
IQR: Interquartile range
OR: Odds ratio
CI: Confidence interval
N.S.: No significant
N.D.: Not detected

## References

[1] Nasri D, Bouslama L, Pillet S, Bourlet T, Aouni M, Pozzetto B (2007). Basic rationale, current methods and future directions for molecular typing of human enterovirus. Expert Rev Mol Diagn.

[2] Simmonds P, Gorbalenya AE, Harvala H, Hovi T, Knowles NJ, Lindberg AM, Oberste MS, Palmenberg AC, Reuter G, Skern T (2020). Recommendations for the nomenclature of enteroviruses and rhinoviruses. Arch Virol.

[3] Pons-Salort M, Parker EP, Grassly NC (2015). The epidemiology of non-polio enteroviruses: recent advances and outstanding questions. Curr Opin Infect Dis.

[4] Berardi A, Sandoni M, Toffoli C, Boncompagni A, Gennari W, Bergamini MB, Lucaccioni L, Iughetti L (2019). Clinical characterization of neonatal and pediatric enteroviral infections: an Italian single center study. Ital J Pediatr.

[5] Abzug MJ (2004). Presentation, diagnosis, and management of enterovirus infections in neonates. Paediatr Drugs.

[6] Solomon T, Lewthwaite P, Perera D, Cardosa MJ, McMinn P, Ooi MH (2010). Virology, epidemiology, pathogenesis, and control of enterovirus 71. Lancet Infect Dis.

[7] Ooi MH, Wong SC, Lewthwaite P, Cardosa MJ, Solomon T (2010). Clinical features, diagnosis, and management of enterovirus 71. Lancet Neurol.

[8] World Health Organization. A guide to clinical management and public health response for hand, foot and mouth disease (HFMD).

[9] Xing W, Liao Q, Viboud C, Zhang J, Sun J, Wu JT, Chang Z, Liu F, Fang VJ, Zheng Y (2014). Hand, foot, and mouth disease in China, 2008–12: an epidemiological study. Lancet Infect Dis.

[10] Holmes CW, Koo SS, Osman H, Wilson S, Xerry J, Gallimore CI, Allen DJ, Tang JW (2016). Predominance of enterovirus B and echovirus 30 as cause of viral meningitis in a UK population. J Clin Virol.

[11] Kadambari S, Okike I, Ribeiro S, Ramsay ME, Heath PT, Sharland M, Ladhani SN (2014). Seven-fold increase in viral meningo-encephalitis reports in England and Wales during 2004–2013. J Infect.

[12] Abedi GR, Watson JT, Nix WA, Oberste MS, Gerber SI (2018). Enterovirus and parechovirus surveillance - United States, 2014–2016. MMWR Morb Mortal Wkly Rep.

[13] Lafolie J, Labbe A, L'Honneur AS, Madhi F, Pereira B, Decobert M, Adam MN, Gouraud F, Faibis F, Arditty F (2018). Assessment of blood enterovirus PCR testing in paediatric populations with fever without source, sepsis-like disease, or suspected meningitis: a prospective, multicentre, observational cohort study. Lancet Infect Dis.

[14] Chen X, Li J, Guo J, Xu W, Sun S, Xie Z (2017). An outbreak of echovirus 18 encephalitis/meningitis in children in Hebei Province, China, 2015. Emerg Microbes Infect.

[15] Xie J, Yang XH, Hu SQ, Zhan WL, Zhang CB, Liu H, Zhao HY, Chai HY, Chen KY, Du QY (2020). Co-circulation of coxsackieviruses A-6, A-10, and A-16 causes hand, foot, and mouth disease in Guangzhou city, China. BMC Infect Dis.

[16] Stecher G, Tamura K, Kumar S (2020). Molecular evolutionary genetics analysis (MEGA) for macOS. Mol Biol Evol.

[17] Zhang H, Zhao Y, Liu H, Sun H, Huang X, Yang Z, Ma S (2017). Molecular characterization of two novel echovirus 18 recombinants associated with hand-foot-mouth disease. Sci Rep.

[18] Cabrerizo M, Díaz-Cerio M, Muñoz-Almagro C, Rabella N, Tarragó D, Romero MP, Pena MJ, Calvo C, Rey-Cao S, Moreno-Docón A (2017). Molecular epidemiology of enterovirus and parechovirus infections according to patient age over a 4-year period in Spain. J Med Virol.

[19] Khetsuriani N, Lamonte-Fowlkes A, Oberst S, Pallansch MA (2006). Centers for disease C, prevention: enterovirus surveillance–United States, 1970–2005. MMWR Surveill Summ.

[20] Cabrerizo M, Diaz-Cerio M, Munoz-Almagro C, Rabella N, Tarrago D, Romero MP, Pena MJ, Calvo C, Rey-Cao S, Moreno-Docon A (2017). Molecular epidemiology of enterovirus and parechovirus infections according to patient age over a 4-year period in Spain. J Med Virol.

[21] Cheng HY, Huang YC, Yen TY, Hsia SH, Hsieh YC, Li CC, Chang LY, Huang LM (2014). The correlation between the presence of viremia and clinical severity in patients with enterovirus 71 infection: a multi-center cohort study. BMC Infect Dis.

[22] Verboon-Maciolek MA, Krediet TG, Gerards LJ, de Vries LS, Groenendaal F, van Loon AM (2008). Severe neonatal parechovirus infection and similarity with enterovirus infection. Pediatr Infect Dis J.

[23] Lin TY, Kao HT, Hsieh SH, Huang YC, Chiu CH, Chou YH, Yang PH, Lin RI, Tsao KC, Hsu KH, Chang LY (2003). Neonatal enterovirus infections: emphasis on risk factors of severe and fatal infections. Pediatr Infect Dis J.

[24] Martin NG, Iro MA, Sadarangani M, Goldacre R, Pollard AJ, Goldacre MJ (2016). Hospital admissions for viral meningitis in children in England over five decades: a population-based observational study. Lancet Infect Dis.

[25] Hsu CH, Lu CY, Shao PL, Lee PI, Kao CL, Chung MY, Chang LY, Huang LM (2011). Epidemiologic and clinical features of non-polio enteroviral infections in northern Taiwan in 2008. J Microbiol Immunol Infect.

[26] Rhoades RE, Tabor-Godwin JM, Tsueng G, Feuer R (2011). Enterovirus infections of the central nervous system. Virology.

[27] Bersani I, Auriti C, Piersigilli F, Dotta A, Diomedi-Camassei F, Di Pede A, Buttinelli G, Danhaive O (2020). Neonatal acute liver failure due to enteroviruses: a 14 years single NICU experience. J Matern Fetal Neonatal Med.

[28] Khetsuriani N, Lamonte A, Oberste MS, Pallansch M (2006). Neonatal enterovirus infections reported to the national enterovirus surveillance system in the United States, 1983–2003. Pediatr Infect Dis J.

[29] Modlin JF (1988). Perinatal echovirus and group B coxsackievirus infections. Clin Perinatol.

[30] Khediri Z, Vauloup-Fellous C, Benachi A, Ayoubi JM, Mandelbrot L, Picone O (2018). Adverse effects of maternal enterovirus infection on the pregnancy outcome: a prospective and retrospective pilot study. Virol J.

[31] Lv XQ, Qian LH, Wu T, Yuan TM (2016). Enterovirus infection in febrile neonates: a hospital-based prospective cohort study. J Paediatr Child Health.

[32] Abzug MJ, Levin MJ, Rotbart HA (1993). Profile of enterovirus disease in the first two weeks of life. Pediatr Infect Dis J.

[33] Muslin C, Mac Kain A, Bessaud M, Blondel B, Delpeyroux F (2019). Recombination in enteroviruses, a multi-step modular evolutionary process. Viruses.

[34] Simmonds P, Welch J (2006). Frequency and dynamics of recombination within different species of human enteroviruses. J Virol.

[35] Nikolaidis M, Mimouli K, Kyriakopoulou Z, Tsimpidis M, Tsakogiannis D, Markoulatos P, Amoutzias GD (2019). Large-scale genomic analysis reveals recurrent patterns of intertypic recombination in human enteroviruses. Virology.

[36] Oberste MS, Maher K, Pallansch MA (2004). Evidence for frequent recombination within species human enterovirus B based on complete genomic sequences of all thirty-seven serotypes. J Virol.

[37] Xiao Y, Rouzine IM, Bianco S, Acevedo A, Goldstein EF, Farkov M, Brodsky L, Andino R (2016). RNA recombination enhances adaptability and is required for virus spread and virulence. Cell Host Microbe.

[38] Xiao Y, Dolan PT, Goldstein EF, Li M, Farkov M, Brodsky L, Andino R (2017). Poliovirus intrahost evolution is required to overcome tissue-specific innate immune responses. Nat Commun.

