# Supplementary material for: Enterovirus B types cause severe infection in infants aged 0–3 months
Source: Virol J. 2023 Jan 9;20:5. doi: 10.1186/s12985-023-01965-9 (PMC9830867; doi:10.1186/s12985-023-01965-9)
Supplement: Supplementary file 1 — Additional file 1: Table S1. Primers used for complete genome amplification of echovirus 11 [file 12985_2023_1965_MOESM1_ESM.docx]

Supplementary table S1. Primers used for complete genome amplification of echovirus 11

| Primer | Sequence (5′-3′) | Nucleotide position^a^ | Polarity |
| --- | --- | --- | --- |
| E11-F1 | TTAAAACAGCCTGTGGGTTG | 1-20 | sense |
| E11-R1 | AAATGTGACCTCCACRTC | 2804-2821 | antisense |
| E11-F2 | GACACCATGCARACCAGRCA | 2594-2613 | sense |
| E11-R2 | TGGRCARCACTCYTCATCACA | 4886-4906 | antisense |
| E11-F3 | GTGTACTCHCTMCCACCAGA | 4541-4560 | sense |
| E11-R3 | CCGCACCGAATGCGGARAAT | 7419-7438 | antisense |
| ^a^Numbering according to the genome of echovirus 11 prototype strain (GenBank accession number: [X80059](http://www.ncbi.nlm.nih.gov/nuccore/X80059)). | | | |
