# Supplementary material for: Enterovirus B types cause severe infection in infants aged 0–3 months
Source: Virol J. 2023 Jan 9;20:5. doi: 10.1186/s12985-023-01965-9 (PMC9830867; doi:10.1186/s12985-023-01965-9)
Supplement: Supplementary file 2 — Additional file 2: Table S2. Clinical characteristics of children with EV infection according to EV species, Guangzhou, China, 2019 [file 12985_2023_1965_MOESM2_ESM.docx]

Supplementary table S2. Clinical characteristics of children with EV infection according to EV species, Guangzhou, China, 2019

|  | EV-A infections, n=66 | EV-B infections, n=49 | *p* |
| --- | --- | --- | --- |
| Age (months) | 16.5 (13.0–26.0) | 0.7 (0.4–1.0) | < 0.001 |
| Male sex | 47 (71.2%) | 28 (57.1%) | N.S. |
| Days of hospitalization | 5 (4–7) | 11 (6–16) | < 0.001 |
| Clinical features |  |  |  |
| Fever (≥38℃) | 62 (93.9%) | 41 (83.7%) | N.S. |
| Rash | 53 (80.3%) | 7 (14.3%) | < 0.001 |
| Tachycardia^a^ | 28 (42.4%) | 12 (24.5%) | 0.046 |
| Lethargy | 0 | 2 (4.1%) | N.S. |
| Coughing | 30 (45.5%) | 7 (14.3%) | 0.001 |
| Diarrhea | 15 (22.7%) | 8 (16.3%) | N.S. |
| Vomiting | 13 (19.7%) | 2 (4.1%) | 0.014 |
| Pruritus | 6 (9.1%) | 0 | N.S. |
| Startle | 12 (18.2%) | 0 | 0.002 |
| Convulsions | 23 (34.8%) | 5 (10.2%) | 0.002 |
| Impaired consciousness | 0 | 7 (14.3%) | 0.006 |
| Hand-foot-mouth disease | 55 (83.3%) | 4 (8.1%) | < 0.001 |
| Pneumonia | 13 (19.7%) | 31 (63.3%) | < 0.001 |
| Aseptic meningitis | 10 (15.1%) | 26 (53.1%) | < 0.001 |
| Encephalitis | 2 (3.0%) | 4 (8.2%) | N.S. |
| Sepsis | 5 (7.6%) | 14 (28.6%) | 0.003 |
| Myocarditis | 3 (4.5%) | 8 (16.3%) | N.S. |
| Gastrointestinal dysfunction | 13 (19.7%) | 10 (20.4%) | N.S. |
| Hepatitis | 0 | 8 (16.3%) | 0.002 |
| Pulmonary edema | 2 (3.0%) | 1 (2.0%) | N.S. |
| Severe infection | 26 (39.4%) | 36 (73.5%) | < 0.001 |
| Number of deaths | 0 | 2 (4.1%) | N.S. |
| ^a^Tachycardia: age 0~3 months old, heart rate ≥ 140 times per minute, 4~12 months old, heart rate ≥ 130 times per minute, 1~3 years old, heart rate ≥ 120 times per minute, 4~7 years old, heart rate ≥ 100 times per minute, 8~14 years old, heart rate ≥ 90 times per minute; EV, enterovirus; N.S.: No significant. | | | |
